# Chimeric antigen receptor T cells targeting cell surface GRP78 efficiently kill glioblastoma and cancer stem cells

**DOI:** 10.1186/s12967-023-04330-0

**Published:** 2023-07-22

**Authors:** Shijie Wang, Wenwen Wei, Yuncang Yuan, Bin Sun, Dong Yang, Nan Liu, Xudong Zhao

**Affiliations:** Department of Targeting Therapy and Immunology and Laboratory of Animal Tumor Models, Cancer Center and National Clinical Research Center for Geriatrics and Frontiers Science Center for Disease-related Molecular Network, West China Hospital, Sichuan University, Chengdu, Sichuan China

**Keywords:** csGRP78, CAR-T cells, GBM, GSCs

## Abstract

**Background:**

Glioblastoma (GBM) is recognized as among the most aggressive forms of brain tumor. Patients typically present with a five-year survival rate of less than 6% with traditional surgery and chemoradiotherapy, which calls for novel immunotherapies like chimeric antigen receptor T (CAR-T) cells therapy. In response to endoplasmic reticulum (ER) stress in multiple tumor cells including GBM, the glucose-regulated protein 78 (GRP78) expression increases and the protein is partially translocated to the cell surface, while it is restricted to the cytoplasm and the nucleus in normal cells.

**Methods:**

In this study, to target the cell surface GRP78 (csGRP78), CAR-T cells based on its binding peptide were generated. In vitro two GBM cell lines and glioma stem cells (GSCs) were used to confirm the localization of csGRP78 and the cytotoxicity of the CAR-T cells. In vivo a GBM xenograft model was used to assess the killing activity and the safety of the CAR-T cells.

**Results:**

We confirmed the localization of csGRP78 at the cell surface of two GBM cell lines (U-251MG and U-87MG) and in GSCs. Co-culture experiments revealed that the CAR-T cells could specifically kill the GBM tumor cells and GSCs with specific IFN-γ release. Furthermore, in the tumor xenograft model, the CAR-T cells could decrease the number of GSCs and significantly suppress tumor cell growth. Importantly, we found no obvious off-target effects or T cell infiltration in major organs following systemic administration of these cells.

**Conclusions:**

The csGRP78 targeted CAR-T cells efficiently kill GBM tumor cells and GSCs both in vitro and in vivo, and ultimately suppress the xenograft tumors growth without obvious tissue injuries. Therefore, our study demonstrates that csGRP78 represents a valuable target and the csGRP78-targeted CAR-T cells strategy is an effective immunotherapy against GBM.

**Supplementary Information:**

The online version contains supplementary material available at 10.1186/s12967-023-04330-0.

## Introduction

Glioblastoma (GBM) is among the most common primary malignant brain tumor in adults, and patients suffer the highest rate of mortality from this form of cancer [1]. Although comprehensive treatment regimens including surgery, chemotherapy with temozolomide, and radiation therapy are used in the clinic to treat this condition, patient prognosis remains poor. Patients diagnosed with GBM have a mean overall survival of only 12–15 months, and the five-year survival rate is less than 6% [2–4]. Thus, there is a pressing need for more effective therapeutic options, including experimental immunological approaches such as chimeric antigen receptor T (CAR-T) cell treatment.

The field of cancer immunotherapy has been re-energized by the application of CAR-T cell therapy, especially in hematological malignancies of the B-cell linage [5–7]. For the treatment of GBM, promising results have been reported in clinical studies using CAR-T cells that target the epidermal growth factor receptor variant III (EGFRvIII), interleukin 13 receptor subunit alpha 2 (IL13Rα2), or human epidermal growth factor receptor 2 (HER2) [8–10]. Recently, clinical trials have begun to evaluate the effectiveness of CAR-T cells targeting human B7 homolog 3 (B7-H3), CD147, or the disialoganglioside GD2 (GD2) against recurrent GBM [11–14]. However, due to variable therapeutic results and side-effects, the use of these approaches for clinical purposes has been suspended. These unfortunate outcomes motivated us to identify novel antigen targets.

Glucose-regulated protein 78 (GRP78), belonging to the heat-shock protein 70 (HSP70) family, resides primarily in the endoplasmic reticulum (ER) and functions as a chaperone during the process of protein folding and assembly in normal cells [15]. ER stress increases in tumor cells as a consequence of dysregulated proliferation, which leads to inadequate blood supply, hypoxia, nutrient deprivation and immune reactions [16]. To adapt these changes, GRP78 levels are increased and the protein is partially relocalized from the ER to the cell surface. Subsequently, GRP78 forms complexes with other proteins on the cell surface to mediate tumor cell signal transduction. For example, in highly metastatic and invasive 1-LN prostate cancers, GRP78 interacts with α_2_-macroglobulin at the cell surface to promote tumor cell proliferation through the ERK1/2 - p38/MAPK - PI3K pathway, and cell survival through the Akt - NF-kB signaling cascade [17]. Additionally, when complexed with Cripto at the cell surface, GRP78 can enhance tumor growth by inhibiting TGF-β signaling [18]. However, the GRP78, which only rests inside the cell, will not transfer in normal cells, and this indicates that csGRP78 on tumor cells may be viable as a target for cancer-cell specific therapy [19, 20].

Indeed, various immunotherapies have been developed to target cell surface GRP78 (csGRP78). Zhao and coworkers recently demonstrated that nanoparticles conjugated with a GRP78 monoclonal antibody and loaded with 5-fluorouracil (5-FU) were selectively delivered to hepatocellular carcinoma cells, and this led to loss of cell adhesion and invasion in vitro [21]. In another study, a GRP78 peptide (WIFPWIQL) conjugated with doxorubicin (DOX) displayed enhanced anticancer activity in colorectal cancer xenografts compared to control studies with free DOX [22]. Finally, CAR-T cells targeting csGRP78 were found to be highly effective in models of acute myeloid leukemia (AML) [23, 24].

GRP78 levels are reported to be upregulated in GBM specimens when compared to control brains, low-grade astrocytomas, and oligodendrogliomas, and elevated GRP78 expression is significantly correlated with poor patient survival [25–27]. Compared to normal cells that typically express GRP78 only at low levels and within the ER, glioma and blood vascular endothelial cells display overexpression of GRP78 and an increase in csGRP78[19, 27, 28]. Therefore, csGRP78 becomes worthy of exploring as a tumor target against GBM and the therapeutic efficacy by CAR-T cells has not been reported. In this study, we have assessed the feasibility of using CAR-T cells targeting csGRP78 as a novel therapeutic paradigm against GBM tumor cells and cancer stem cells in vitro and in vivo.

## Materials and methods

### Cell lines and culture

U-251MG and U-87MG cell lines were purchased from China Infrastructure of Cell Line Resources (Kunming, China). They were cultured in DMEM supplemented with 10% FBS, 100 U/mL penicillin and 100 mg/mL streptomycin (Life Technologies, USA). The GSC-3# and GSC12# cell lines, isolated from primary glioblastoma tissues, were previously described [29]. U-251MG cells and U-87MG cells were transduced with lentivirus vectors expressing luciferase, which were named U251-luc and U87-luc respectively. U-251MG-derived CSC spheres were cultured from U251-luc cells in the medium containing serum-free DMED/F12 supplemented with 20 ng/mL EGF, 20 ng/mL bFGF and 1x B27 (all from Life Technologies, USA). All cells were grown in a humidified incubator with 5% CO_2_ at 37 ℃.

### Plasmid design and lentivirus packaging

The GRP78-targeted second-generation CAR constructs was previously prepared by our lab [24]. They contained the GRP78-binding peptide Pep42, followed by a CD8 hinge spacer, a CD8 transmembrane domain, a 4-1BB sequence, and a CD3ζ endo-domain linked to the far-red fluorescence mKate2 sequence by the 2 A self-cleaving peptide. These sequences were cloned into a lentiviral expressing plasmid named Pep42-BBZ. Mock-BBZ lacked the Pep42 sequence, but was otherwise the same as the Pep42-BBZ plasmid. The lentiviral packaging plasmids pMD2.G and pCMVΔ8.91 (Addgene, USA) as well as either Pep42-BBZ or mock-BBZ were co-transduced into HEK293T cells at a ratio of 4:10:20. Lentivirus was collected at 48 and 72 h post-transfection for concentration by centrifuging, and the titer of the concentrated lentivirus was calculated.

### Generation of CAR-T cells

Peripheral blood was from healthy donors and T cells were isolated by RosetteSep™ Human T Cell Enrichment Cocktail (STEMCELL, Canada). T cells was cultured in advanced 1640 (Life Technologies, USA) supplemented with 10% FBS, penicillin, streptomycin, 1x Glutamax (Gibco, USA) and 200 U/mL IL-2 (PeproTech, USA). After T cells were activated for 72 h by CD3/CD28 Dynabeads (Life Technologies, USA), they were transfected with lentivirus containing either the Pep42-BBZ plasmid or mock-BBZ plasmid at MOI = 30 in the presence of lentiboost (SIRION biotech, Germany). CAR expression (mKate2 ratio) was subsequently assessed 96 h later by flow cytometry and used in the following experiments. For proliferation experiment, T cells from the three groups were cultured until day 17 post transduction.

### Immunofluorescence

Prior to the IF staining, CSCs were adhered to plates overnight using laminin (Gibco, USA). For surface staining of GRP78, cells were washed with PBS for 5 min and incubated with GRP78 primary antibodies (PA1-014 A, Invitrogen, USA) at 1:200 dilution for 1 h at room temperature. Cells were washed with PBS for 5 min, fixed by 4% paraformaldehyde for 15 min, and blocked by 10% goat serum in 0.1% BSA, 0.1% Tween-20 and PBS for 1 h. Cells were then incubated with Cy3-conjuncted goat anti-rabbit secondary antibody (A10520, Invitrogen) at 1:1000 dilution for 1 h at room temperature in the dark. Cells were washed three times with PBS for 5 min and stained with 5 µg/mL DAPI for 10 min. Finally, coverslip were extracted, mounted in mounting medium (Polysciences, USA), covered with a slide and stored at 4 ℃ for imaging. Specificity of staining was confirmed by the inclusion of negative controls that were stained with secondary antibodies alone.

### Vector copy number assessment by QRT-PCR

The average integrated vector copy number (VCN) per cell of Pep42 CAR-T cells was assessed by SYBR Green QRT-PCR method (reagent cat NO. A25742, Thermo Fisher). Genomic DNA was extracted from 1 × 10^6^ Pep42 CAR-T cells using isopropanol sedimentation and 100 ng DNA was used per QRT-PCR reaction. To generate a standard curve, serial dilution of Pep42-CAR plasmid standard (1, 10^− 1^, 10^− 2^, 10^− 3^, 10^− 4^, 10^− 5^ and 10^− 6^ ng/ul) and the corresponding VCN (copies/ul) were used as horizontal axis and the corresponding ct values were used as vertical axis. QRT-PCR was performed using the following conditions: 2 min for 50 ℃, 10 min for 95 ℃, followed by 40 cycles of denaturation at 15 s for 95 ℃ and annealing/extension of 1 min for 60 ℃. The primer sequences were as follows:

Forward: 5’ TGGCGGCTATGTGAGA 3’;Reverse: 5’ GTTTGCAGTAAAGGGTGAT 3’.

### Cytotoxicity assays in vitro

A luciferase-based assay was used to determine the cytotoxicity of CAR-T cells towards U-251MG and U-87MG GBM cells, as well as U-251MG CSCs. Prior to the cytotoxicity assays, CSCs were adhered to plates overnight using laminin (Gibco, USA). Approximately 2000 target cells were seeded into wells of a 96-well plate. Control (non-transduced), mock-BBZ and Pep42-BBZ CAR-T cells were co-cultured with target cells in 96-well plates at (E:T) ratios of 1:1, 2:1, 4:1 and 8:1. All effector cells used in the assay were based on total T cells, including CAR^+^ T cells and CAR^−^ T cells after transduction in each group. The supernatant was removed 20 h post-culturing, and stored at − 80 ℃ in preparation for use in ELISA assays. Cells were then lysed and 30 µL luciferase substrate (Promega, USA) added prior to luminescence being measured on a microplate reader. All cytotoxicity ratios were calculated based on the control (non-transduced) cytotoxicity. All the killing assays were repeated by three independent experiments from three different donors.

To determine the cytotoxicity of CAR-T cells towards the GSC3# and GSC12# cell lines, a Cell-Mediated Cytotoxicity Fluorometric Assay Kit (BioVision, USA) was used. Briefly, 4 × 10^4^ CFSE-labeled target cells (GSC3# or GSC12#) were seeded into wells of a 96-well plate. Subsequently, control (non-transduced), mock-BBZ, and Pep42-BBZ CAR-T cells (effector cells) were added to each well at an E:T ratio of 4:1. All effector cells used in the assay were based on total T cells, including CAR^+^ T cells and CAR^−^ T cells after transduction in each group. After 20 h of co-culturing, all cells were collected, stained with 7-aminoactinomycin D (AAD) and quantified by flow cytometry. All the killing assays were repeated by three independent experiments from three different donors.

### ELISA

The effector cells, control (non-transduced), mock-BBZ CAR, or Pep42-BBZ CAR-T cells were co-cultured with target cells for 20 h at an E:T ratio of 4:1 before the supernatant was assessed for IFN-γ levels by enzyme-linked immunosorbent assay kits (Invitrogen, USA) according to the manufacturer’s instructions.

### Xenograft mouse model

Six to eight-week-old female severely immune-deficient NOD-Prkdcem26Cd52Il2rgem26Cd22/Nju (NCG, T001475) mice were purchased from the GemPharmatech Co., Ltd of Nanjing (China) and housed in specific pathogen-free environment at the Laboratory Animal Center of West China Hospital. 5 × 10^5^ U251-luc cells in 0.9% saline containing 30% Matrigel (BD Bioscience) were injected subcutaneously into the right flanks. When tumor diameters reached ~ 2 mm five days later, mice were divided into three groups randomly. They received one of the following injections of T cells intravenously into their tail veins: 1 × 10^7^ control (non-transduced) T cells, 1 × 10^7^ mock-BBZ CAR-T cells, or 1 × 10^7^ Pep42-BBZ CAR-T cells. Tumor growth was monitored once a week using an in vivo imaging software (IVIS) system (Lumina Xr, PerkinElmer, USA). Bioluminescent signals were quantified using Living Image Software (Caliper Life Science). Mice were sacrificed when the tumor volume reached approximately 2000 mm^3^. All protocols were approved by the animal ethics committee of the West China Hospital, SICHUAN University.

### Immunohistochemical analysis and H&E staining

Tumors, hearts, livers, spleens, lungs, kidneys and brains from the mock-BBZ CAR and Pep42-BBZ CAR groups were embedded in paraffin, and then sliced into sections with a thickness of 4 μm. Sections were deparaffinized, rehydrated, and processed for antigen retrieval. H&E staining was performed on heart, liver, spleen, lung, kidney, and brain tissue sections to observe their structure. The tumor, liver and lung tissue sections were blocked with 10% goat serum and incubated with the indicated primary antibodies at 4 ℃ overnight. Cy3-conjuncted goat anti-rabbit secondary antibody (A10520, Invitrogen), FITC-conjuncted goat anti-rabbit secondary antibody (F-2765, Invitrogen), and Cy3-conjuncted goat anti-mouse secondary antibody were used (A10521, Invitrogen). The primary antibodies used were as follows: anti-GRP78 antibody (PA1-014 A, Invitrogen), anti-CD3ζ antibody (ET1607-20, Huabio), anti-NESTIN antibody (sc23927, Santa Cruz), anti-CD4 antibody (ET1606-31, HUABIO), and anti-CD8 antibody (ET1609-52, HUABIO).

### Statistical analyses

All statistical analyses were conducted with GraphPad Prism 7.0 statistical software. Data was presented as mean ± SD. Statistical differences between two groups were analyzed using Student t tests with Welch correction. Statistical differences among three or more groups were analyzed by one-way ANOVA. Statistical significance was defined as *P ≤ 0.05, **P ≤ 0.01, ***P ≤ 0.001.

## Results

### CAR-T cells can be engineered to recognize csGRP78 and kill GBM cell lines

**Fig. 1:**
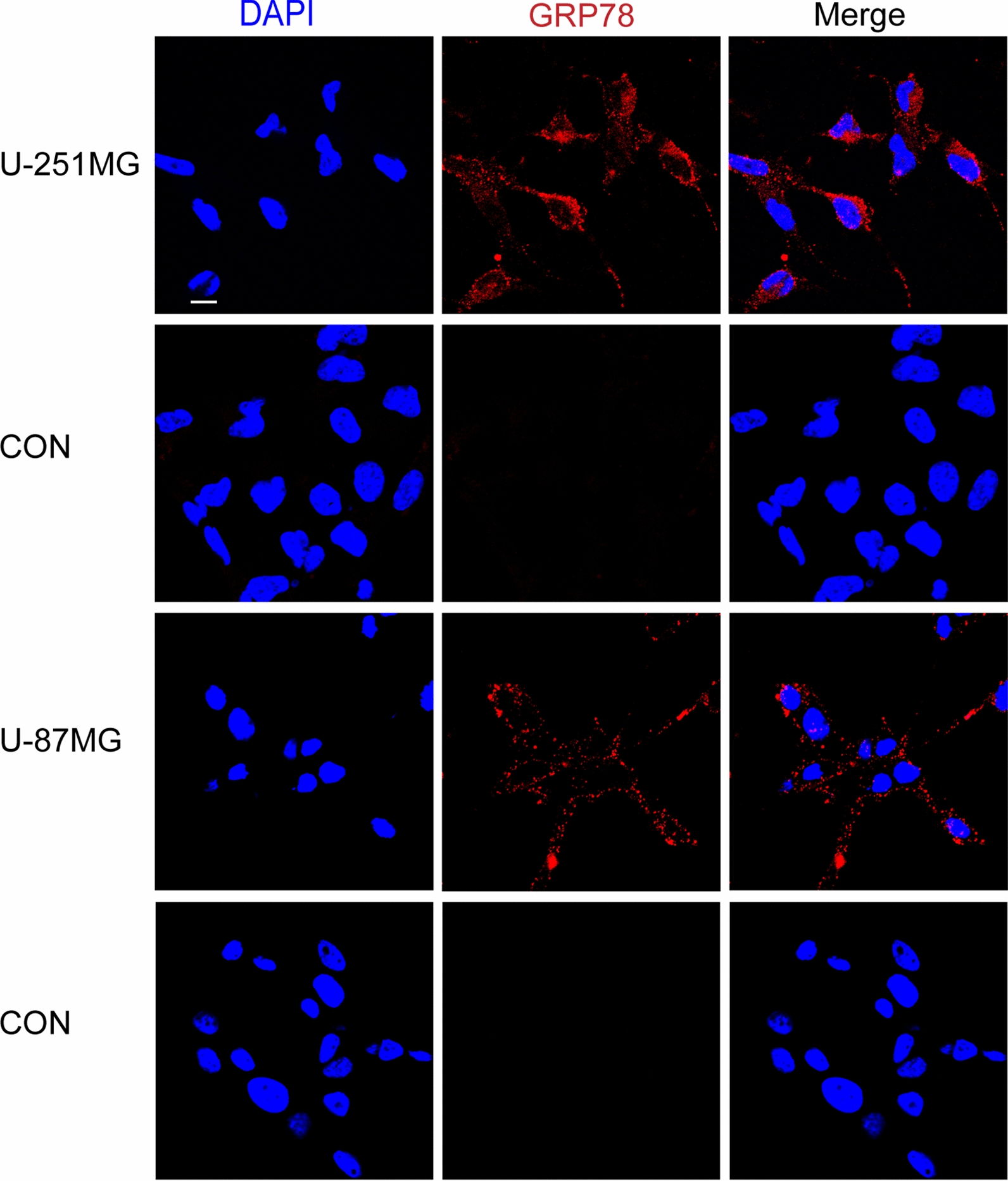
GRP78 expression on human glioma cell lines. Surface immunofluorescence staining of two human glioma cell lines (U-251MG and U-87MG) showing the localization of GRP78 at the cell surface. Cells were stained with GRP78 polyclonal antibodies and Cy3-conjuncted secondary antibodies. Control cells were stained with only Cy3-conjuncted secondary antibodies. Scale bar = 10 μm

**Fig. 2:**
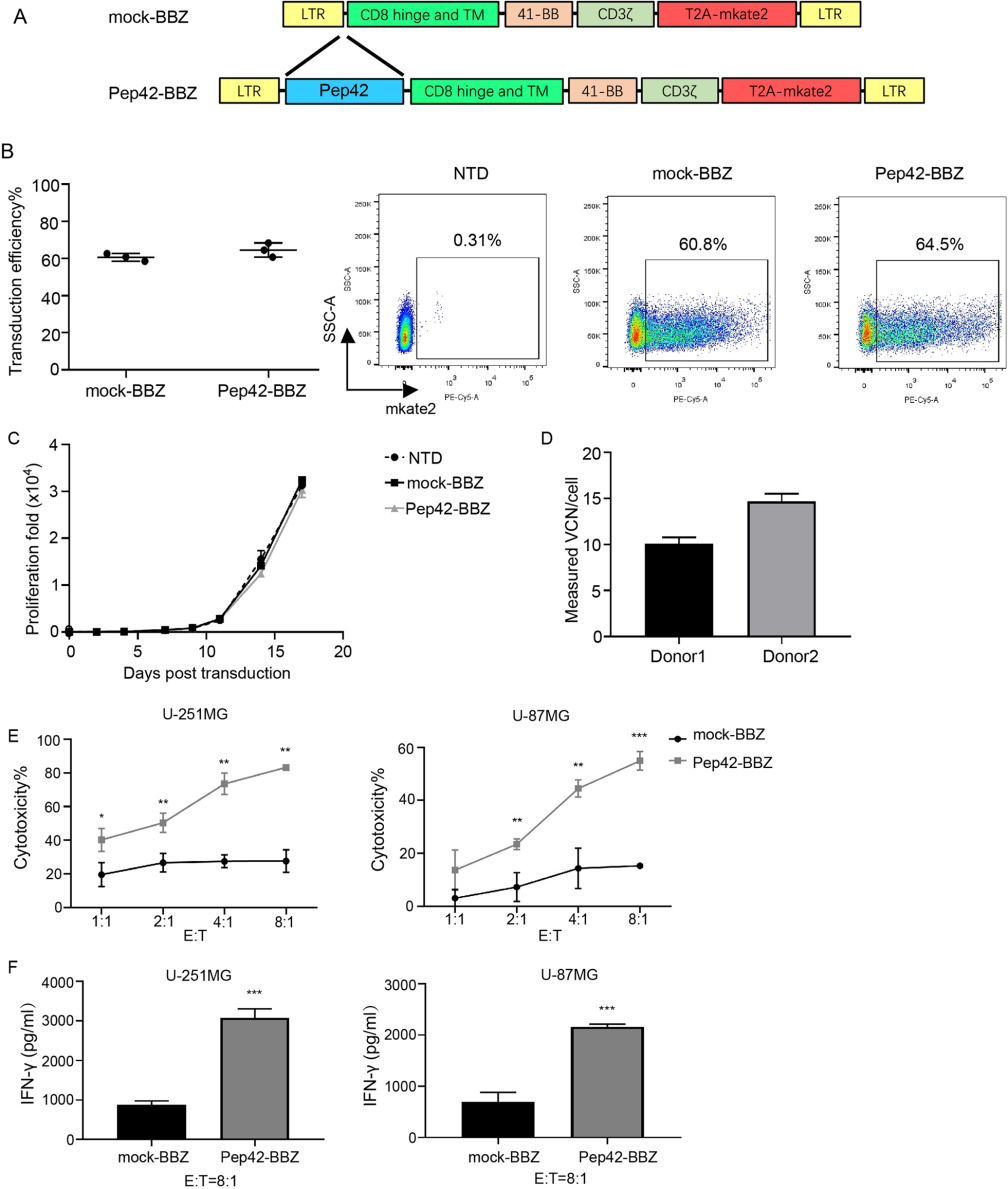
Pep42-BBZ CAR-T recognized GRP78 and kill glioma cell lines. **A** Schematic showed the structure of mock-BBZ and Pep42-BBZ CARs. **B** Quantification of the percentage of CAR-positive T cells 96 h post transduction detected by flow cytometry. Left: Statistics of transduction efficiency of Pep42-BBZ CAR-T group and mock-BBZ CAR-T group from three healthy donors. Right: Representative images from one donor of flow cytometry. The results were from three independent experiments. NTD = non-transduced T cell controls. **C** The proliferation fold change of the three groups of T cells from day 0 to day 17 post lentiviral construct transduction. **D** Measured VCN per cell of Pep42 CAR-T cells derived from two of the three healthy donors above. Cells were assessed 96 h post transduction and the transduction MOI and efficiency were the same as the above. **E** Cytotoxicity ratios of mock-BBZ and Pep42-BBZ CAR-T cells to U-251MG and U-87MG cells at increasing E:T ratios of 1:1, 2:1, 4:1 and 8:1. CAR-T cells were co-cultured with target cells for 20 h. Results were calculated based on the lysis ratio of NTD T cells to the target cells at every E:T ratio. **F** IFN-γ release levels from the supernatant of cells co-cultured at an E:T ratio of 8:1 quantified by ELISA. All effector cells used in the cytotoxicity assay were based on total T cells in each group. Data presented as the mean volume ± SD, * P < 0.05, ** P < 0.01 *** P < 0.001

To detect the cell surface localization of GRP78, we performed surface immunofluorescence (IF) on two human GBM cell lines (U-251MG and U-87MG). As shown in Fig. 1, GRP78 was strongly expressed on the cell surface of both cell lines. To target csGRP78 on these cells, the GRP78-binding peptide Pep42 was incorporated into a second-generation CAR sequence, expressing a 4-1BB domain and a CD3ζ domain (Pep42-BBZ), fused with the far-red fluorescence mkate2 as a marker. As a negative control, a mock-BBZ was the same as the Pep42-BBZ construct but lacked the Pep42 sequence (Fig. 2A). To determine whether T cells can obtain stable CAR expression from separate donors retaining individual differences, T cells isolated from the peripheral blood of healthy donors were transfected with the Pep42-BBZ or mock-BBZ plasmids. The expression of the mKate2 marker was assessed 96 h post-transfection using flow cytometry. It was shown that the average transduction efficiency of Pep42-BBZ CAR in T cells from three different healthy donors was 62.6 ± 1.9%, which was comparable with the control group mock-BBZ CAR of 60.63 ± 1.03% (Fig. 2B). To observe the impact of CAR transduction and CAR-T fratricide, T cells were maintained for 17 days post transduction. The proliferation fold of Pep42-BBZ CAR-T cells was comparable with mock-BBZ CAR-T cells and NTD T cells, with no significant decrease (Fig. 2C). Further, it is important to assess the vector copy number (VCN) as it is recommended by the FDA for the release of the CAR-T final products especially when they are considered on clinical usage. The average VCN of Pep42 CAR-T cells 96 h post-transfection derived from two donors was about 10/cell and 15/cell respectively (Fig. 2D).

To determine the cytotoxicity of the Pep42-BBZ CAR-T cells (effector cells), they were co-cultured with U-251MG or U-87MG cells (target cells) at gradient effector to target (E:T) ratios ranging from 1:1 to 8:1. Compared with mock-BBZ CAR-T cells, Pep42-BBZ CAR-T cells significantly killed both cell lines in a dosage-dependent manner (Fig. 2E). To further confirm the activation of T cells and specificity of csGRP78 targeting, the supernatant from cells co-cultured with an 8:1 ratio was collected for analysis of interferon γ (IFN-γ) release. Pep42-BBZ CAR-T cells generated significantly more IFN-γ compared to mock-BBZ CAR-T cells (Fig. 2F). Together, these data suggested that Pep42-BBZ CAR-T cells can specifically recognize csGRP78 and kill csGRP78-positive GBM cell lines.

### Pep42-BBZ CAR-T cells display cytotoxicity to glioma stem cells in vitro

**Fig. 3:**
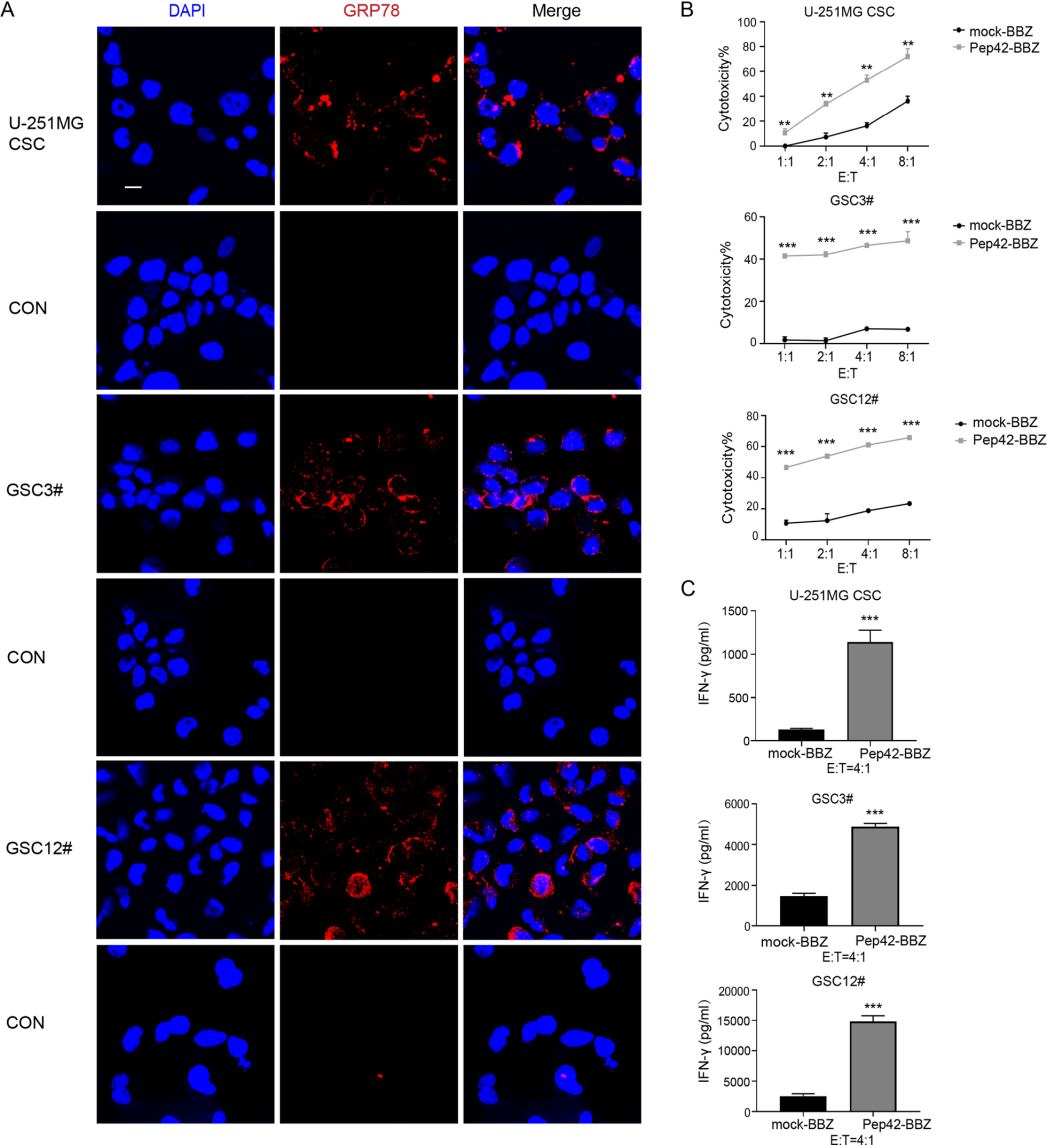
Cytotoxicity of Pep42-BBZ CAR-T cells towards GSCs in vitro. **A** Immunofluorescence (IF) staining of U-251MG CSC, GSC3# and GSC12# for csGRP78. Thirty-six hours prior to IF, cells were adhered to laminin-coated coverslips. The staining procedure was the same as for U-251MG cells. Scale bars = 10 μm. **B** Quantification of the relative cytotoxicity of CAR-T cells to the three GSCs lines. Target cells were cultured on laminin-coated coverslips overnight before co-culturing. CAR-T cells were co-cultured with target cells for 20 h at increasing E:T ratios of 1:1, 2:1, 4:1, and 8:1. All effector cells used in the assay were based on total T cells in each group. The cytotoxicity ratio was calculated based on flow cytometry results as described in the "Materials and methods" section. **C** ELISA-mediated quantification of IFN-γ release levels in the by supernatants from cells co-cultured at an E:T ratio of 4:1. Data presented as the mean volume ± SD, ** P < 0.01 *** P < 0.001

Previous reports have demonstrated that csGRP78 can function in maintaining glioma stem cells (GSCs) stemness [30]. To determine if Pep42-BBZ CAR-T cells can target GSCs, we used three cells lines previously generated in our laboratory: U-251MG-derived cancer stem cell (CSC) spheres (U-251MG CSC), and two human primary GBM tissue-derived cells lines cultured in vitro (GSC3# and GSC12#) [31]. The stem cell markers NESTIN, SOX2 and CD133 were used to confirm the stemness of these cell lines by QRT-PCR, which was consistent with the previous report [31], together with the IF staining of NESTIN expression in the three stem cells (Supplementary Fig. 1). The localization of GRP78 at the cell surface was confirmed by IF (Fig. 3A). When cultured at increasing E:T ratios, Pep42-BBZ CAR-T cells significantly killed the three GSCs in a dosage-dependent manner (Fig. 3B), and caused significantly elevated levels of IFN-γ release compared to mock-BBZ CAR-T cells (Fig. 3C) at the E:T ratio of 4:1. Thus, the Pep42-BBZ CAR-T cells displayed cytotoxicity to GSCs cultured in vitro.

### Pep42-BBZ CAR-T cells show antitumor activity in vivo

**Fig. 4:**
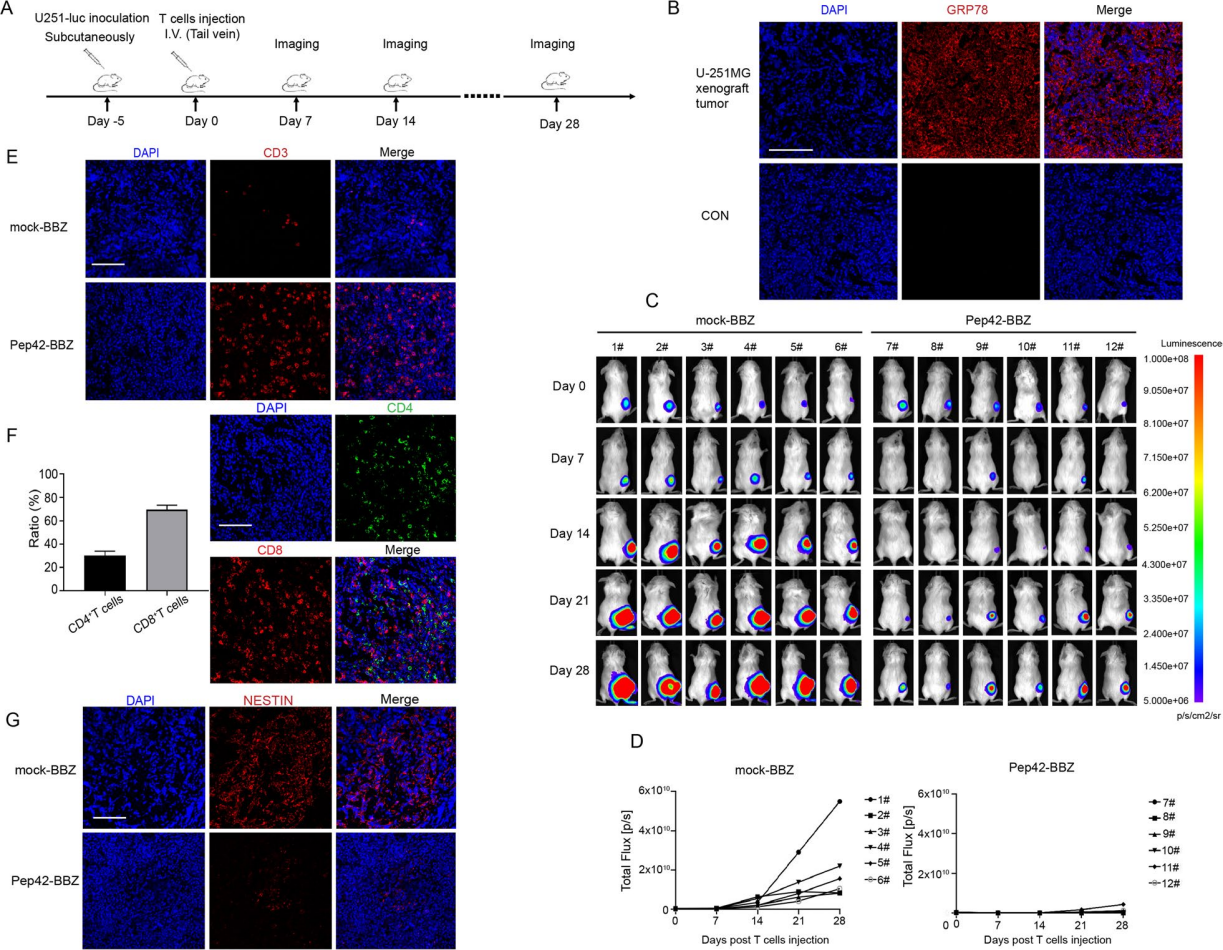
Pep42-BBZ CAR-T cells showed antitumor activity in vivo. **A** Schematic in the top panel illustrates the experiment design for the U-251MG xenograft tumors. NCG mice was inoculated subcutaneously with 5 × 10^6^ luciferase-expressing U-251MG cells. Five days later mock-BBZ and Pep42-BBZ CAR-T cells (1 × 10^7^ per mouse) were injected intravenously into tail veins. The tumors growth was monitored once a week. **B** A representative image of csGRP78 staining of xenograft tumor sections by IF. Five days after tumor cells inoculation, tumor tissues were extracted and cell surface IF was performed for GRP78. Control was stained without GRP78 antibody. **C** The tumor bioluminescence images by IVIS imaging. **D** Tumor bioluminescence intensity curves for the two groups. **E** Representative CD3 staining of tumor sections by IF. Five-days post injection, 3 tumors from each group were extracted, embedded in paraffin, sectioned, and stained with CD3ζ antibody coupled with Cy3-conjunctated secondary antibodies. Two representative images are included from each group. **F** The statistics and representatives of CD4 and CD8 staining on Pep42-BBZ CAR-T treated tumor sample sections. The numbers of FITC labeled-CD4 and Cy3 labeled-CD8 were calculated and performed for percentage counting. **G** Representative NESTIN staining of tumor sections by IF. Five-days post injection, 3 tumors from each group were extracted, embedded in paraffin, sectioned, and stained with NESTIN antibodies coupled with Cy3-conjunctated secondary antibodies. Two representative images from each group are shown. Scale bar = 100 μm

To examine the antitumor efficacy of Pep42-BBZ CAR-T cells in vivo, we generated a GBM tumor xenograft model. Severely immunodeficient NCG mice were subcutaneously inoculated with U-251MG cells stably expressing luciferase as shown in the schematic (Fig. 4A). Five-days post-inoculation when tumor diameters reached ~ 2 mm, extra xenograft tumors were dissected to confirm the antigen target expression. We observed csGRP78 was abundantly expressed within the xenograft tumor sections (Fig. 4B). Then the tumor-bearing mice were randomly divided into two groups and injected with either mock-BBZ CAR-T cells or Pep42-BBZ CAR-T cells intravenously into their tail veins. The bioluminescence intensity was measured once per week until the tumor volumes reached ~ 2000 mm^3^. Although tumors remained in both groups, tumor growth was significantly decreased in the Pep42-BBZ CAR-T group when compared with mock-BBZ CAR-T group (Fig. 4C, D). Tumor sections from 3 mice in each group were collected on day five post-injection and stained for the T cell marker CD3. Consistent with our data on tumor growth, tissue taken from the Pep42-BBZ CAR-T group displayed abundant CD3 staining, while only scarce staining was observed in mock-BBZ CAR-T tissue (Fig. 4E). Further, the average percentage of tumor-infiltrated CD4^+^ T cells and CD8^+^ T cells were 30% and 70% respectively, which was consistent with the principle of more CD8^+^ T cells to kill tumor cells (Fig. 4F). Moreover, we also tested the ability of CAR-T cells to kill GSCs by analyzing the expression of the GSC marker NESTIN. IF staining on tumor sections revealed high levels of NESTIN expression in the mock-BBZ CAR-T group, but very low levels in the Pep42-BBZ CAR-T group (Fig. 4G). Taken together, these data suggested that Pep42-BBZ CAR-T cells can kill U-251MG tumor cells and GSCs within tumor xenografts, resulting in the suppression of tumor growth.

### Pep42-BBZ CAR-T cells display high levels of specificality and safety

**Fig. 5:**
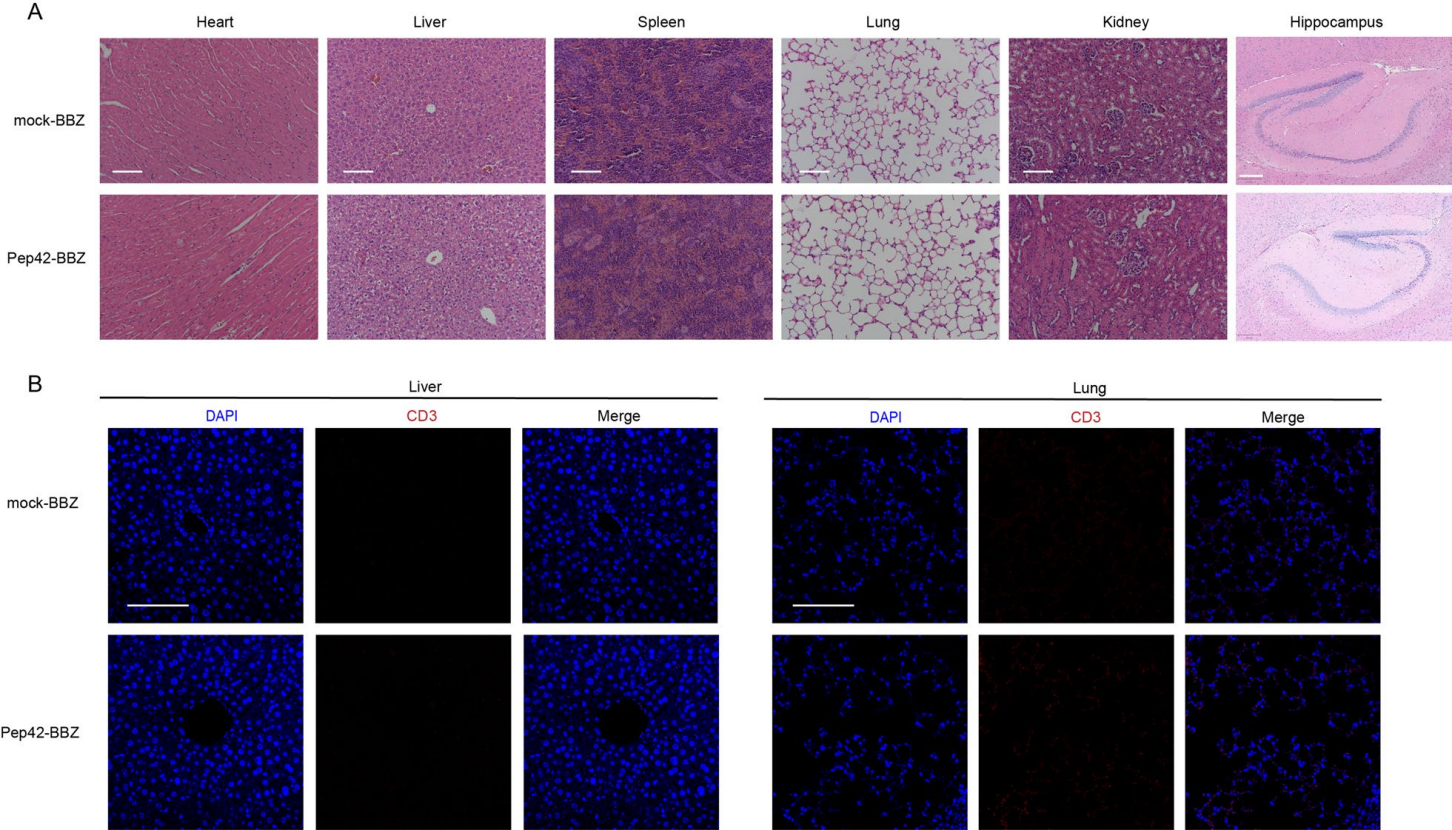
Specificity and safety of Pep42-BBZ CAR-T cells. **A** Representative images of H&E stained hearts, livers, spleens, lungs, kidneys, and hippocampi sections from both mock-BBZ and Pep42-BBZ CAR-T groups. Two groups of CAR-T cells were injected intravenously into tails veins of tumor-bearing mice (n = 3). After five days, organs were extracted and made into paraffin sections. Scale bars for heart, liver, spleen, lung, and kidney images = 100 μm; scale bar for hippocampi images = 200 μm. **B** Representative IF staining of livers and lungs with CD3ζ antibodies coupled with Cy3-conjunctated secondary antibodies by IF staining. Scale bars = 100 μm

To investigate the specificity and safety of Pep42-BBZ CAR-T cells, we collected tissues from six main organs and performed H&E staining to examine tissue organization and structure. The hearts, livers, spleens, lungs, kidneys, and hippocampus of mice injected with either mock-BBZ CAR-T or Pep42-BBZ CAR-T cells all appeared normal with no obvious irregularities (Fig. 5A). Furthermore, lung and liver sections were also stained with a CD3ζ antibodies to determine if T cell accumulation was occurring in these organs with abundant blood supply. Few T cells could be observed in the liver sections and only background signal was observed in the lungs (Fig. 5B). Thus, it appeared that Pep42-BBZ CAR-T cells might home to tumor sites with high-specificity and safety.

## Discussion

Although csGRP78 is known to positively correlate with GBM malignancy and negatively correlate with patient survival, the use of csGRP78-targeted CAR-T cells to treat GBM tumors has not previously been reported. In the current study, we confirmed the expression and localization of GRP78 on the surface of two GBM cell lines and GSCs. Furthermore, we demonstrated that Pep42-BBZ CAR T cells could kill both GBM cell lines and GSCs in vitro and release IFN-γ. Similarly, these csGRP78-targeted CAR-T cells could kill tumor cells, reduce the number of GSCs, and ultimately suppress GBM tumor growth in vivo in xenografts.

Several clinical trials have been completed for CAR-T cell therapy against GBMs, including those targeting EGFRvIII [32–34], Her2 [8, 35] and IL13Rα2 [36]. Additional clinical trials are ongoing for CAR-T cells targeting EphA2 [35, 37], GD2 [38]and B7-H3 [11, 39]. These trials have all shown variable degrees of remission in both preclinical and clinical assessments. However, loss or decrease of antigen in tumor cells has become the main obstacles for EGFRvIII’s further study [32], and overlapping of the antigen expression on normal tissues has caused off-target side-effects in patients receiving CAR-T cells targeting IL13Rα2 [40] and EphA2 [41]. A polyclonal antibody directed against csGRP78 has been shown to induce apoptosis in melanoma (A375) and prostate cancer cells (1-LN, DU145) through the up-regulation of p53, inhibition of NF-kappa B1 and NF-kappa B2 activation, and suppression of Ras/MAPK and PI3K/Akt signaling [27, 42, 43]. Knockdown of GRP78 by siRNA can suppress the proliferation of GBM cell lines through attenuating the Akt and ERK1/2 pro-survival pathways [44]. Overall, csGRP78 is strongly correlated with malignancy [15] and its loss or decrease on tumor cells seems unlikely. Previous studies have shown that csGRP78 is barely detectable in the normal organs of U-87MG tumor-bearing mice [19]. In human prostate cancer and AML xenografts, targeted therapies can specifically recognize tumor cells, while the main organs and hematopoietic progenitors survive [20, 23]. Therefore, overlapping expression of csGRP78 also seems unlikely.

Numbers of reports have confirmed the over-expression of GRP78 in tumor cells and human GBM tissues mainly at mRNA and protein levels [25, 26, 28], without the examination of its surface localization especially for the U-251MG cell line. In the current study, we performed the surface IF staining for GRP78 without rupturing the cell membranes, such that GRP78 can clearly be seen on the membrane of U-251MG and U-87MG cells. Our findings are consistent with previous publications for the U-87MG cell line [19, 27].

The far-red fluorescence protein mKate2 was fused with CAR by T2A on the plasmid and the proportion of mKate2 marker represented CAR expression here. However, mKate2 does not completely reflect the true expression of CAR. Self-cleaving 2 A peptides are promising candidates for the production of multicistronic vectors. 2 A is usually used in CAR constructs in recent reports [45, 46]. Although the translation process will be inhibited at the 2 A cleavage site, the subsequent protein will not be affected in most cases. While sometimes the self-cleavage mechanism fails which leads to the formation of fused protein. Sometimes the translation stops at the 2 A site and eventually only the first protein is synthesized [47]. Based on the mechanisms above the expression of CAR will be more than mKate2 rather than less. Therefore, we used mKate2 as a marker to reflect the expression of CAR by flow cytometry.

Due to their unlimited self-renewal and tumorigenic capacities, GSCs are considered the initiating cells of GBMs [48], and they play a crucial role in the malignant progression [30]. Thus, GSCs are key therapeutic targets and CAR-T cells can be engineered to target them. CD133-specific CAR-T cells were shown to kill GSCs and slightly prolong the survival of glioma-bearing mice [49], while GD2-specific CAR-T cells can prolong survival in glioma nerve stem cell-derived tumor-bearing mice [50]. Huynh and coworkers reported that GRP78 expression is significantly elevated in cultured cancer stem cell spheres compared to their parental counterparts [51]. Downregulating GRP78 expression or targeting csGRP78 can strongly suppress the self-renewal, migration and invasion of GSCs, and reduce the expression of three transcription factors (STAT3, NF-κB and C/EBPβ) that are essential for maintaining GSCs [30]. We have confirmed the expression of GRP78 on the cell surface of GSCs, including those contained in U-251MG and U-87MG cultured cell spheres and patient-derived GSC3# and GSC12#. Moreover, our in vitro and in vivo results demonstrate that Pep42-BBZ CAR-T cells can effectively kill GSCs and reduce the number of GSCs marked by NESTIN within xenograft tumor sections, which is consistent with the tumor growth results. We speculate that decreasing the number of GSCs and inhibiting their self-renewal ability will ultimately retard tumor growth.

Although we observed CAR-T cell-mediated killing of U-251MG tumor xenografts, we did not achieve complete elimination of tumors and saw variable tumor recurrence. We identify three reasons for this failure to attain complete tumor elimination. Firstly, the capacity of Pep42-BBZ T cells to kill U-251MG cells or GSCs is limited to approximately 60% by inherent causes, including the incomplete transduction of activation signals in T cells after the recognition between CARs and antigens. Secondly, the ability of CAR-T cells to infiltrate the tumor mass may be restricted by suppressive cells and molecules within the immunosuppressed microenvironment [49]. Thirdly, the CAR-T cells may not persist long enough or sufficiently proliferate in the blood [23]. Accordingly, the structure of CARs may need to be optimized to improve their proliferative capacity and to enhance the activation levels of T cells. Combination therapy using CAR-T cells together with traditional treatments may be another means of boosting their efficacy.

Our H&E staining studies revealed no significant structural changes in major organs after the administration of csGRP78-targeting CAR-T cells. This is consistent with the previous report, which found that csGRP78-targeting CAR-T could suppress AML without inducing hematopoietic progenitor cell toxicity [23]. Furthermore, upon systemic administration of Pep42-BBZ CAR-T cells, we observed no obvious infiltration into organs with abundant blood flow. Consistent with this, Arap and coworkers reported that GRP78 binding peptide motifs specifically target tumor cells without colonizing major organs in a human prostate cancer xenograft model [20]. Also, we blast the sequence of GRP78 the longest transcript variants from Mus musculus and the Homo sapiens, showing that it is conserved between the two species. Although these murine models may only represent a surrogate for human studies, they provide important information about the safety of csGRP78-targeting CAR-T therapy and demonstrate their utility as an invaluable preclinical model.

## Conclusions

In conclusion, we demonstrated that csGRP78 was a valuable antigen and the targeted CAR-T cells efficiently kill GBM tumor cells and stem cells both in vitro and in vivo. GRP78 was confirmed to be localized on the cell surface of the two GBM cell lines and stem cells. csGRP78-targeted CAR-T cells killed the tumor cells in an antigen-dependent manner and specifically released IFN-γ. In the GBM tumor xenograft model, the CAR-T cells infiltrated into the tumor tissues, decreased the number of GSCs, and ultimately suppressed tumor growth. Further, main organs were kept away from T cells enrichment and displayed no obvious injuries.

## Supplementary Information

**Additional file 1: Fig. S1.** Glioma stem cellmarkers confirmation. **A** Immunofluorescence (IF) staining of U-251MG CSC,GSC3# and GSC12# for NESTIN. Thirty-six hours prior to IF, cells were adheredto laminin-coated coverslips. Scale bars = 50 μm. **B** QRT-PCRquantification of NESTIN, SOX2, and CD133 expression in U-251MG CSC, GSC3# andGSC12#. The relative expression calculation was based on U-251MG. Datapresented as the mean volume ± SD, * P < 0.05, ** P < 0.01 *** P <0.001.

## Data Availability

The datasets of this work are available from the corresponding author on reasonable request.

## Abbreviations

GBM: Glioblastoma
GRP78: Glucose-regulated protein 78
csGRP78: Cell surface GRP78
ER: Endoplasmic reticulum
GSCs: Glioma stem cells
CAR: Chimeric antigen receptor
VCN: Vector copy number
EGFRvIII: Epidermal growth factor receptor variant III
IL13Rα2: Interleukin 13 receptor subunit alpha 2
HER2: Human epidermal growth factor receptor 2
B7-H3: B7 homolog 3
GD2: Gangliosides 2
HSP70: Heat-shock protein 70
5-FU: 5-fluorouracil
DOX: Doxorubicin
IF: Immunofluorescence
AML: Acute myeloid leukemia
IFN-γ: Interferon-γ
IVIS: In vivo imaging software
