# Supplementary material for: Chimeric antigen receptor T cells targeting cell surface GRP78 efficiently kill glioblastoma and cancer stem cells
Source: J Transl Med. 2023 Jul 22;21:493. doi: 10.1186/s12967-023-04330-0 (PMC10362566; doi:10.1186/s12967-023-04330-0)

**Additional file 1: Fig. S1**. Glioma stem cell markers confirmation. **A** Immunofluorescence (IF) staining of U-251MG CSC, GSC3# and GSC12# for NESTIN. Thirty-six hours prior to IF, cells were adhered to laminin-coated coverslips. Scale bars = 50 μm. **B** QRT-PCR quantification of NESTIN, SOX2, and CD133 expression in U-251MG CSC, GSC3# and GSC12#. The relative expression calculation was based on U-251MG. Data presented as the mean volume ± SD, * P < 0.05, ** P < 0.01 *** P < 0.001.


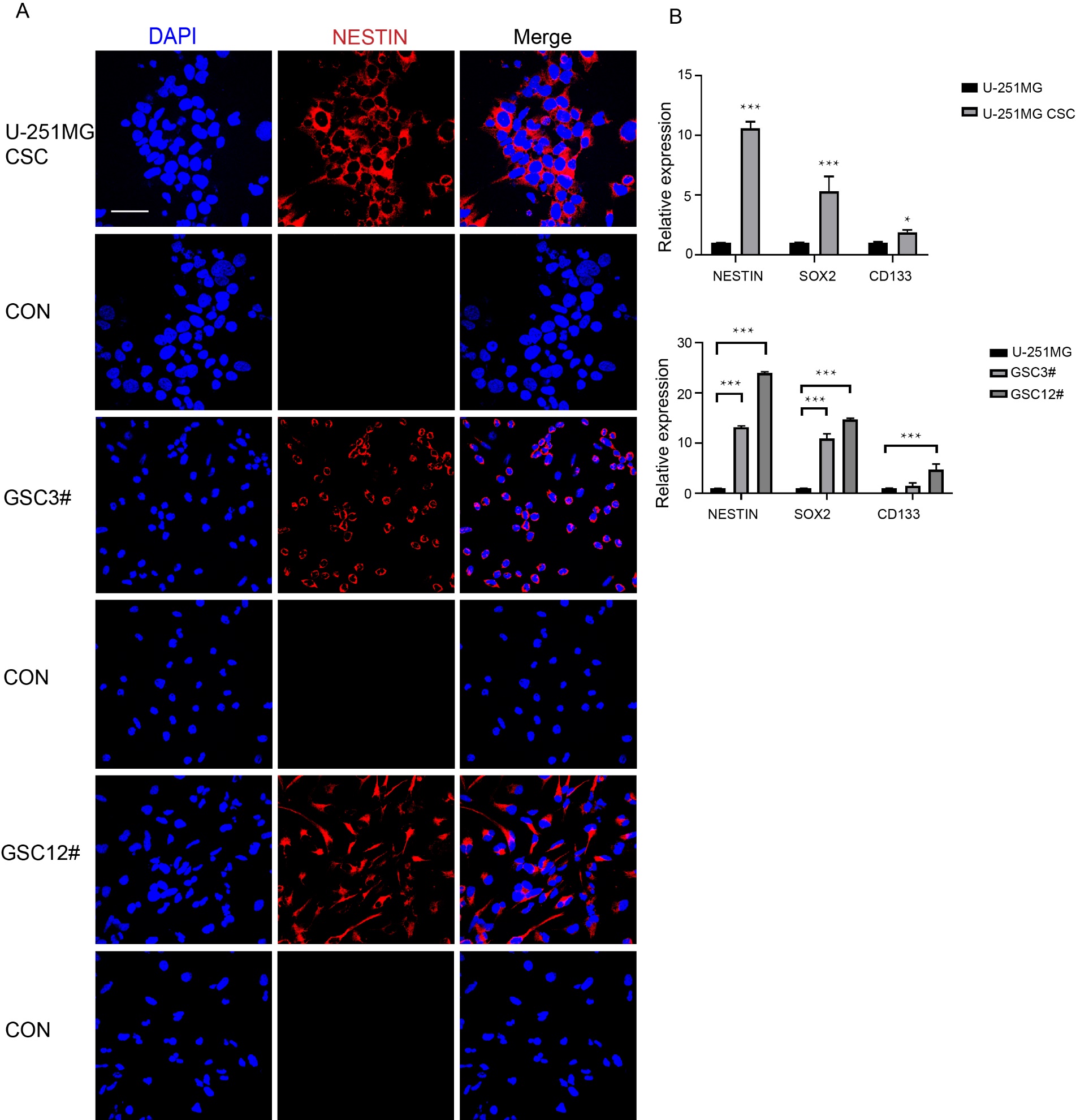

Supplement: Supplementary file 1 — Additional file 1: Fig. S1. Glioma stem cellmarkers confirmation. A Immunofluorescence (IF) staining of U-251MG CSC,GSC3# and GSC12# for NESTIN. Thirty-six hours prior to IF, cells were adheredto laminin-coated coverslips. Scale bars = 50 μm. B QRT-PCRquantification of NESTIN, SOX2, and CD133 expression in U-251MG CSC, GSC3# andGSC12#. The relative expression calculation was based on U-251MG. Datapresented as the mean volume ± SD, * P < 0.05, ** P < 0.01 *** P <0.001. [file 12967_2023_4330_MOESM1_ESM.docx]
